# EcoCert-V2X: Risk-Aware Adaptive Cooperation for Communication-Efficient V2X Cooperative Perception

**DOI:** 10.3390/s26196025

**Published:** 2026-09-23

**Authors:** Malek Alrashidi, Wajih Abdallah

**Affiliations:** Department of Computer Science, Applied College, University of Tabuk, Tabuk 71491, Saudi Arabia

**Keywords:** cooperative perception, V2X, connected vehicles, intelligent transportation systems, communication efficiency, risk-aware AI, sensor networks, operational carbon, non-inferiority, resilient perception

## Abstract

Cooperative perception can improve the situational awareness of connected and autonomous vehicles by exchanging complementary sensing information among nearby agents. However, systematic multi-vehicle cooperation also increases vehicle-to-everything (V2X) communication demand, processing overhead, and operational resource use. This study introduces EcoCert-V2X, a risk-aware adaptive cooperation framework that selects the level of cooperative feature acquisition according to action-admissibility scores derived from the pre-communication operating context. Rather than transmitting all available peer information systematically, EcoCert-V2X selects among a frozen set of cooperation actions while accounting for perception quality, communication volume, latency, graphics processing unit (GPU) action energy, modeled communication energy, and operational carbon. The framework is evaluated using a leakage-controlled development, calibration, and locked confirmatory protocol on the V2V4Real benchmark, which contains real-world vehicle-to-vehicle light detection and ranging (LiDAR) observations. Across 1846 confirmatory samples from eight scenarios and 30 paired operational seeds, EcoCert-V2X achieved a scenario-balanced task risk of 0.6513 compared with 0.6433 for full cooperation. The resulting risk difference of +0.0080 satisfied the prespecified non-inferiority criterion, with a one-sided 95% upper bound of +0.0188 below the margin of 0.03. Communication volume decreased by 14.94%, while operational carbon decreased by 4.07%. The observed GPU action energy reduction remained statistically inconclusive, and the absolute risk target of 0.5730 was not reached. Under six frozen packet erasure and signal-to-noise ratio (SNR) perturbation conditions, aggregate non-inferiority was preserved, although localized scenario–seed sensitivity remained. These results show that risk-aware adaptive V2X cooperation can reduce communication demand while preserving relative cooperative perception performance in connected-vehicle environments.

## 1. Introduction

The development of intelligent transportation systems increasingly depends on connected and resource-aware mobility infrastructures capable of supporting safe transportation while limiting the computational and environmental burden of digital services. Cooperative connected and automated mobility has therefore emerged as a major component of next-generation intelligent transportation systems, combining artificial intelligence (AI), vehicle-to-everything (V2X) communication, edge computing, and distributed sensing [1,2,3]. In this scenario, cooperative perception is critical because an ego vehicle is constrained by limitations such as sensor range, occlusion, unfavorable viewing geometry, and incomplete line-of-sight information. V2X-assisted collaborative perception can extend the effective sensing field and improve environmental awareness beyond what individual vehicles can achieve by exchanging sensing information and learned features with nearby vehicles and roadside infrastructure [2,3,4]. The growing maturity of this field is also reflected in real-world benchmarks such as DAIR-V2X and V2V4Real, which provide vehicle–infrastructure and vehicle–vehicle data for cooperative perception under practical traffic conditions [5,6].

However, these perception gains incur additional communication and computational costs. The cooperative systems must send out their observations, neural features, or final outputs over wireless connections characterized by fluctuating bandwidth and reliability due to varying mobility and channel conditions. The process of fusing information at the early and intermediate stages is capable of providing ample supplementary knowledge, but too much sharing could lead to increased volumes of communication and unnecessary computations. These limitations have motivated the development of communication-efficient techniques such as Where2comm, which transmits only spatially informative features, together with recent methods that explicitly prioritize communication utility [7,8]. This raises the central question of how much cooperation is required for a given operating context, rather than whether cooperation is beneficial in general. In the case of scaling smart regions, the transmission of irrelevant information makes use of bandwidth, computing capacity, and energy with no guarantee of increasing operational efficiency.

Communication efficiency alone is insufficient to ensure practical deployment. V2X system links are influenced by packet loss, interruptions, delays, synchronization failures, localization uncertainty, and the heterogeneity of sensing configurations. The issues V2X-ViT takes into account include the heterogeneity of technologies, uncertainties in pose, and asynchronous data exchange [4]. At the same time, the experiments conducted by the designers of interruption-aware cooperative perception have shown that lost collaborative data might be one of the reasons for the drop in performance of the perception process [9]. Hence, different studies indicate that loss-affected communication, time misalignment, and the problem of network dependency are major challenges [2,3]. This requirement is of particular significance when favorable average communication savings are of little use in safety-critical mobile AI if they lead to uncontrolled failures in hard situations.

A second challenge concerns the environmental cost of increasingly complex vehicle intelligence. Modern autonomous systems simultaneously integrate multiple sensors, V2X interfaces, and complex AI models. The requirements of the sensors, communication, and inference systems substantially increase energy consumption as it is required to repeatedly compute and exchange large tensors between the cooperating systems.

The idea of approximate edge AI in autonomous driving states that it is necessary to consider computation and communication jointly and to avoid optimizing prediction accuracy in isolation [10]. Green Edge AI emphasizes that energy-aware data collection and reasoning are the main requirements of sustainable intelligent operations close to the data sources [11]. This perspective motivates a broader assessment of sustainable mobility. In particular, the sustainability evaluation should go beyond the number of bytes transferred and include computation energy, communication energy, latency, and carbon footprint in operation.

Reducing the consumption of resources raises a parallel question about reliability. Specifically, it creates a need for having a reliable method for adjusting cooperation. Predictive uncertainty estimation provides a number of methods for expressing the reliability of outputs produced by machine learning systems [12]. At the same time, the idea of conformal risk control goes beyond the limits of calibration as it defines the way of controlling more general loss functions [13]. The presented approaches are relevant in the case of cooperative perception since communication can be regarded as a method for adaptive data acquisition: if the local evidence is sufficient, no further peer information is needed, while complex cases necessitate higher levels of cooperation. However, uncertainty-aware prediction, communication efficiency, and sustainable edge technologies have largely developed as separate research directions. Researchers often put emphasis on one of these directions, focusing either on the accuracy of perception or bandwidth, the robustness of the communication systems, or energy efficiency.

Because relatively few studies integrate communication, perception, statistical risk qualification, and sustainability within a single operational framework, EcoCert-V2X is proposed to address this gap. The framework distinguishes several predefined cooperation levels instead of treating full cooperation as the only operating mode, allowing the selected level to depend on the current context and operating situation. Moreover, the approach avoids characterizing sustainability through a single proxy such as bandwidth or energy consumption alone, since the EcoCert-V2X methodology jointly considers multiple operational indicators, including communication volume, latency, GPU action energy, modeled communication energy, and operational carbon.

This work makes four main contributions. First, we present a risk-aware AI policy that dynamically selects among a set of predefined V2X cooperation actions while retaining full cooperation as a deterministic fallback when lower-cost actions are not sufficiently supported. Second, we adopt a leakage-controlled development, calibration, and confirmatory protocol in which the feature space, candidate actions, operating threshold, and statistical decision rules are fixed before the final evaluation. Third, we conduct an integrated sustainability-oriented assessment that considers communication volume together with latency, GPU action energy, modeled communication energy, and operational carbon. Finally, robustness is evaluated under predefined packet erasure and SNR perturbations, with additional scenario- and seed-level sensitivity analyses used to identify localized performance degradation.

The locked confirmatory analysis indicates that EcoCert-V2X can reduce communication demand while preserving relative perception performance under the prespecified non-inferiority criterion. The results also distinguish supported operational carbon savings from statistically inconclusive GPU action energy effects, and identify localized robustness limitations.

## 2. Related Work

### 2.1. V2X Cooperative Perception and Multi-Agent Fusion

Cooperative perception has progressed from the exchange of final detections toward feature-level and bird’s-eye-view (BEV) fusion architectures in which spatially distributed agents construct a richer representation of the environment. Recent surveys classify this field according to sensing modality, fusion level, communication topology, and vehicle–infrastructure cooperation [14,15]. Transformer-based methods such as V2X-ViT [4] and CoBEVT [16] have demonstrated that attention mechanisms can effectively aggregate multi-agent spatial information. Subsequent studies focused increasingly on deployment constraints. CoAlign improves collaborative 3D detection in the presence of pose errors through agent-object consistency [17], BM2CP combines LiDAR and camera information and supports cooperation under modality degradation [18], and HEAL extends collaborative perception toward open heterogeneous systems in which new agent types can be incorporated without retraining the entire cooperative architecture [19]. Together, these developments show a transition from homogeneous fusion under ideal assumptions toward heterogeneous and deployment-oriented cooperative perception.

The development of highly realistic datasets has made such a change possible. Apart from DAIR-V2X and V2V4Real [5,6], TUMTraf-V2X provides synchronized onboard and roadside camera–LiDAR observations to allow for cooperative detection and tracking [20], whereas RCooper puts its emphasis on the large-scale collaborative use of roadside sensing across intersections and corridors [21]. These efforts allow for evaluation not just in terms of applications dealing with vehicle formations, but also under occlusions, complicated maneuvers, the presence of various viewing angles, and spatially distributed sensing, which adds to the complexity of systems under development.

A complementary perspective is provided by practical AI-based advanced driver-assistance systems (ADAS) relying primarily on local vehicle sensing. Guerrero-Contreras et al. [22] investigated a cost-effective computer vision and deep-learning solution for inter-vehicle distance estimation, including deployment on resource-constrained hardware. Local perception avoids dependence on V2X communication but remains limited by the sensing range, visibility, and occlusions of the ego vehicle. Cooperative V2X perception can complement such local information with observations from other agents, although at the cost of additional communication and computation. These two approaches are therefore complementary: efficient local perception can provide the primary vehicle-level information, while adaptive cooperation can supply additional information when local evidence is insufficient.

### 2.2. Communication-Efficient Cooperative Perception

Communication efficiency has consequently become a central research direction. Where2comm [7] introduced spatial confidence-driven feature selection to suppress task-irrelevant transmissions. How2comm later combined information-aware feature filtering with delay compensation to address communication redundancy and temporal misalignment jointly [23]. ERMVP uses hierarchical feature sampling while also addressing localization uncertainty [24], whereas CodeFilling replaces dense feature transmission with compact codebook indices and information-demand-driven message selection [25]. These methods demonstrate that a considerable portion of collaborative information can be compressed or omitted without necessarily eliminating the perception benefit of cooperation.

Communication optimization has also been studied from a networking perspective. CCPAV jointly prioritizes cooperative information and cellular V2X resource allocation [26], while scalable collective-perception mechanisms reduce redundant messaging and channel load [27]. Learning-based V2V communication has been used to compress intermediate features and characterize the effects of communication impairments [28]. EdgeCooper explicitly incorporates network conditions into cooperative LiDAR perception, further demonstrating that perception and communication decisions should not be optimized independently [29]. More recently, Wu et al. [30] provided a comprehensive perspective on the evolution of intelligent vehicular communications from LLM-enabled systems toward agentic and embodied AI, emphasizing the increasing integration of perception, communication, and control in next-generation V2X environments. Collectively, these studies establish the perception–bandwidth trade-off as a fundamental property of V2X cooperation. Most existing methods, however, optimize a feature mask, compression ratio, partner set, or network allocation mainly according to perception performance and communication cost. They generally do not formulate the amount of cooperation as a small, interpretable set of operating actions governed by a frozen task-risk admissibility rule.

### 2.3. Robustness Under Imperfect V2X Conditions

Real-world cooperative perception is affected by localization errors, temporal asynchrony, fading, packet loss, interruptions, and imperfect channel-state information. CoAlign directly targets pose-induced spatial misalignment [17], while CoBEVFlow compensates asynchronous collaborative information through estimated BEV motion fields [31]. Interruption-aware cooperative perception models missing collaborative information explicitly [9]. Channel-aware studies further show that intermediate representations respond differently to wireless impairments, and that adaptive weighting can improve robustness under Rician fading and imperfect channel-state information [28,32]. FreeAlign derives inter-agent spatial-temporal alignment from perceptual geometry rather than relying entirely on external localization and clock devices [33]. Recent empirical evidence has further shown that communication interference and online operating conditions can materially affect V2X cooperative perception behavior [34]. At the network level, resource scheduling studies for C-V2X also highlight the importance of interference, resource contention, and communication prioritization when multiple vehicles compete for wireless resources [35]. In addition to its focus on road-based cooperative perception, the latest smart region research has covered the reliable use of autonomous mobile systems in unfavorable sensing and communication conditions. For instance, Semenov et al. developed an intelligent navigation scheme for UAV operation considering limited sensory input or low sensor reliability [36]. Other researchers have looked at UAV communications in terms of security and robustness using adaptive protection for ADS-B data [37]. While the studies mentioned above are concerned with aerial transportation, the need for the reliable operation of autonomous mobile technology has been emphasized within the context of smart-city development. Even though the studies discussed above contributed to improving robustness, average values have still been used to assess performance, which leaves a separate issue for investigation, namely, whether the adaptation policy preserves the predetermined relationship with full cooperation both at the aggregate level and in localized scenario–seed combinations. This issue is addressed in EcoCert-V2X through frozen perturbation conditions and a fixed non-inferiority margin, instead of defining robustness only through average changes in detection results.

### 2.4. Risk Calibration and Sustainability-Aware AI

Uncertainty quantification provides a complementary foundation for deciding when an AI system should trust a prediction or acquire additional information. Modern conformal methods offer distribution-free predictive guarantees for black box models [38], while conformal risk control extends this principle from prediction set coverage to more general loss functions [13]. The Learn-then-Test framework further formulates calibration as a multiple-testing problem, and provides finite-sample control of prespecified risks without refitting the underlying predictive system [39].

These works provide methodological motivation for the separation between development, calibration, and confirmatory evaluation in EcoCert-V2X; however, the proposed framework does not instantiate a conformal prediction procedure and does not claim a distribution-free conformal finite-sample guarantee.

These developments are relevant to adaptive cooperative perception because peer information can be interpreted as an additional information-acquisition resource whose use should depend on the expected risk associated with a lower-cost action.

Simultaneously, Green AI and sustainable AI research suggests model quality assessment should involve computational capability as well as environmental costs [10,11].

Recent reviews emphasize the need to report energy use, resource efficiency, and carbon impacts alongside model performance [40,41]. However, communication-efficient V2X perception and Green AI remain only loosely connected because bandwidth savings are often treated as the primary efficiency criterion, while computational energy, communication energy, latency, and operational carbon are rarely assessed jointly. The literature therefore reveals a gap at the intersection of cooperative perception, communication-efficient information acquisition, statistical risk qualification, robustness, and operational sustainability. EcoCert-V2X addresses this intersection by treating the cooperation level as a risk-aware AI decision and evaluating the resulting frozen policy jointly in terms of task risk, communication volume, latency, GPU action energy, modeled communication energy, operational carbon, and robustness.

## 3. Proposed EcoCert-V2X Framework

### 3.1. Framework Overview

EcoCert-V2X is designed as a risk-aware adaptive cooperation layer for vehicle-to-vehicle collaborative perception. Rather than transmitting a fixed amount of intermediate information for every scene, the framework determines how much peer information should be acquired from a discrete set of communication actions. Full cooperation remains available whenever lower-cost cooperation is not sufficiently supported by the learned action admissibility model. Communication efficiency is therefore treated as an explicit operating decision rather than as a fixed property of the perception architecture.

The perception backbone is instantiated on the V2V4Real real-world V2V benchmark [6] using the CoST collaborative-perception implementation [42] (official implementation: https://github.com/buaa-colalab/CoST. accessed on 19 September 2026). CoST integrates observations from various agents and from various points in time into a joint spatio-temporal representation, which serves as a basis for the investigation of communication reduction while maintaining temporal context. EcoCert-V2X operates the detector in the same way as in the aforementioned scenario—the perception backbone remains frozen, while only the cooperation action changes. This distinction between the two notions allows one to track the variations in communication, latency, energy, and perception risk to the action policy.

When the action is selected during inference, ego-side and pre-cooperation cognitive descriptors are derived and passed to the action-admissibility ensemble. The controller opts for the least costly action that receives the required support based on the order *A*_0_, *A*_1_, and *A*_3_; if there is no admissible action, *A*_5_ is selected deterministically. Next, the chosen spatial budget is applied to the representation and the information is processed using the frozen model for 3D detection. Additionally, the system is able to store task risk, operational resources such as transmission volume, latency, GPU action energy and modeled communication energy information. The overall EcoCert-V2X workflow, including the adaptive cooperation decision path and the separate operational evaluation path, is illustrated in Figure 1.

### 3.2. Adaptive Cooperation Action Space

Let *x_i_* denote the pre-communication context associated with sample *i* and let *a_i_* denote the selected cooperation action. Development initially characterizes six ordered actions spanning no peer-feature transmission to full cooperation—

(1) $$A=\{A_{0},\;A_{1},\;A_{2},\;A_{3},\;A_{4},\;A_{5}\}$$

Each action corresponds to a fixed fraction of the available peer spatial feature grid. The lattice is shown in Table 1. All six actions were measured during development to characterize the perception resource response surface. The final frozen controller retains *A*_0_, *A*_1_, and *A*_3_ as candidate resource-reducing actions and *A*_5_ as the full-cooperation fallback; *A*_2_ and *A*_4_ remain development-only characterization points, and are not eligible for confirmatory reselection.

The frozen payload is 1024 bytes per spatial cell. If *n_i_* is the number of collaborating peers, *C_a_* is the number of cells assigned to action a, and *b_c_* = 1024 bytes/cell, transmitted data are computed exactly as

(2) $$B_{i}(a)=n_{i}\;C_{a}\;b_{cell}$$

(3) $$Q_{i}(a)=8\;B_{i}(a)$$

where *B_i_*(*a*) and *Q_i_*(*a*) denote transmitted bytes and bits, respectively. Communication cost is therefore monotonic by construction across the action lattice. Unlike unconstrained feature masking, every policy decision corresponds to an interpretable operating level with an exactly measurable communication budget.

### 3.3. Distance-Weighted Perception Risk

EcoCert-V2X requires a task-level risk quantity that can be associated directly with each cooperation action. The frozen metric is a distance-weighted 3D miss risk computed from the standard post-processed 3D detections. The official risk routine uses a three-dimensional intersection-over-union threshold of 0.70, a horizontal perception range of $\left[-102.4,-38.4,102.4,38.4\right]$ m, and a maximum distance normalization constant $d_{max}=109.3633$ m.

For each ground-truth object o in sample i of scenario s, the 3D box center is computed as the arithmetic mean of its eight corner coordinates. Let $\left(x_{sio},y_{sio}\right)$ denote the horizontal coordinates of this center. Its horizontal distance from the ego vehicle is

$$d_{sio}=\sqrt{x_{sio}^{2}+y_{sio}^{2}}.$$

The frozen distance-dependent object weight is then defined as

$$w_{sio}=\frac{1}{1+d_{sio}/d_{max}},\;\;d_{max}=109.3633\;m.$$

This monotonically decreasing weighting gives greater contribution to nearby ground-truth objects while retaining a positive contribution from more distant objects. Let $G_{si}$ denote the set of ground-truth objects in sample i. The weighted ground-truth mass is

$$g_{si}=\sum_{o\in G_{si}}w_{sio},$$

and the weighted missed mass under cooperation action a is

$$m_{si}\left(a\right)=\sum_{o\in G_{si}}w_{sio}\;I\;\left[o\;\mathrm{is}\;\mathrm{unmatched}\;\mathrm{under}\;\mathrm{action}\;a\right].$$

Predictions are processed by the frozen one-to-one matching rule in descending confidence order; for each prediction, the unmatched ground-truth object with the highest 3D IoU is selected when ${IoU}_{3D}\geq 0.70$.

For an individual sample i belonging to scenario s, the sample-level task-risk quantity used for action labeling is *R_i_*(*a*) = *m_si_*(*a*)/*g_si_*, when *g_si_* > 0, with the frozen empty-ground-truth convention applied otherwise. This sample-level quantity is used to construct the action-admissibility labels. It should be distinguished from the scenario-level risk *R_s_*(*a*), which is obtained by aggregating weighted missed and ground-truth masses over all samples in scenario s, and from the scenario-balanced primary estimand *R*(*a*).

For scenario s, the scenario-level distance-weighted miss risk is therefore

(4) $$R_{s}\left(a\right)=\frac{\sum_{i\in s}m_{si}\left(a\right)}{\sum_{i\in s}g_{si}}.$$

When a scenario contains no weighted ground-truth mass, the frozen convention assigns zero risk for that empty ground-truth case. Because driving scenarios contain different numbers of frames and objects, directly pooling all samples would allow large scenarios to dominate the final measure. EcoCert-V2X therefore uses the scenario-balanced primary estimand,

(5) $$R\left(a\right)=\frac{1}{S}\sum_{s=1}^{S}R_{s}\left(a\right).$$

where S is the number of scenarios. This estimand is kept identical across calibration, confirmatory evaluation, robustness analysis, and formal inference.

The frozen task-risk estimand focuses on missed ground-truth objects and does not directly penalize false-positive detections. It should therefore be interpreted as a miss-oriented measure of perception risk rather than as a complete detection quality metric.

### 3.4. Risk-Aware AI Admissibility Selector

The decision layer estimates whether a lower-cost cooperation action is acceptable before that action is used. The initial pre-communication context interface contains 21 features derived without executing a reduced-cooperation action. A second feature extractor adds 153 deterministic descriptors: 96 spatial confidence features, 24 spatial entropy features, 24 spatial dispersion features, and 9 peer geometry features. This produces 174 candidate context descriptors. During development, the 174 candidate descriptors were aligned with the fixed input schema of the action admissibility models, yielding the final ordered 165-feature representation. The exact names and ordering of these 165 frozen features are preserved in the shared reproducibility package, and the execution pipeline verifies consistency between the frozen feature order and the selector checkpoints before prediction. This feature lock was completed before calibration or confirmatory access, and no calibration or confirmatory outcome was used to alter the feature composition or ordering.

At the action-labeling stage, a lower-cost action *A_k_* is labeled admissible relative to *A*_5_ when its sample-level task risk does not exceed the corresponding *A*_5_ sample-level task risk by more than the prespecified margin *δ* = 0.03, i.e.,


$$R_{i}(A_{k})\leq R_{i}(A_{5})+\delta .$$

This relative-admissibility criterion is used to learn whether the current context supports a communication-reducing action without requiring the selector to predict the exact risk value. Development subsequently restricts the operational candidate set to *A*_0_, *A*_1_, and *A*_3_, while *A*_5_ remains the fallback.

The action-admissibility models are implemented using HistGradientBoostingClassifier from scikit-learn 1.9.0 with the frozen 165-dimensional pre-communication feature representation. Training uses 5297 samples from 22 training scenarios, while development evaluation uses 752 samples from five separate scenarios. Scenario-level bootstrap weights are applied during training and subsequently balanced between the admissible and non-admissible classes. For each candidate action, three independently seeded models are trained using a learning rate of 0.05, 200 maximum iterations, 31 maximum leaf nodes, a minimum of 30 samples per leaf, L2 regularization of 1.0, and no early stopping.

Table 2 reports only the classifier families retained in the frozen operational selector. *A*_2_ and *A*_4_ were development-only action levels used for action-response characterization, and were not included in the final operational selector.

For each operational candidate a in {*A*_0_, *A*_1_, *A*_3_}, three independently seeded frozen HistGradientBoosting classifiers produce positive-class probabilities *p_a_*^(*j*)^(*x_i_*). The frozen operational selector comprises nine HistGradientBoosting models, with three independently seeded models for each of *A*_0_, *A*_1_, and *A*_3_. The nine serialized checkpoints occupy 8.96 MiB in total, corresponding to approximately 1.00 MiB per model. Each model contains, on average, approximately 12,200 tree nodes and 6200 leaf nodes. These values characterize the memory and structural computational footprint of the selector independently of the frozen cooperative perception backbone. Their arithmetic mean forms the action admissibility score

(6) $${\bar{p}}_{a}\left(x_{i}\right)=\frac{1}{3}\;\sum_{j=1}^{3}p_{a}^{\left(j\right)}\left(x_{i}\right)$$

The final decision rule is hierarchical. Given a frozen threshold *τ*, the controller examines *A*_0_, then *A*_1_, then *A*_3_. The first candidate whose mean admissibility probability reaches *τ* is selected; otherwise the controller returns to *A*_5_,

(7) $$\pi_{\tau }\left(x_{i}\right)=\left\{\begin{array}{cc} A_{0} & if\;{\bar{p}}_{A_{0}}\;\geq \;\tau \\ A_{1} & if\;A_{0}\;fails\;and\;{\bar{p}}_{A_{1}}\;\geq \;\tau \\ A_{3} & if\;A_{0}\;and\;A_{1}\;fail\;and\;{\bar{p}}_{A_{3}}\;\geq \;\tau \\ A_{5} & otherwise \end{array}\right.$$

This ordering gives priority to the smallest admissible communication budget. The *A*_5_ branch is deterministic and prevents the method from forcing communication reduction when no lower-cost action receives sufficient support.

### 3.5. Calibration and Operating-Point Lock

After development, the action models and feature schema are applied to an independent calibration partition containing 1056 samples from five scenarios. The threshold set is fixed before calibration as

(8) T={0.20, 0.30, 0.40, 0.50, 0.60, 0.70, 0.80, 0.90, 0.95}

Full cooperation *A*_5_ defines the calibration reference. Let *R_cal_*(*A*_5_) denote its scenario-balanced calibration risk and let *δ* = 0.03 be the prespecified non-inferiority allowance. The calibration-derived absolute risk target is

(9) $$\alpha_{cal}=R_{cal}\left(A_{5}\right)+\delta$$

With *R_cal_*(*A*_5_) = 0.543035, the frozen value is $\alpha_{cal}$ = 0.573035. All nine thresholds are evaluated using the same action order, risk estimand, and resource definitions. Exactly three thresholds (0.60, 0.70, and 0.80) satisfy all prespecified feasibility gates. The frozen automatic rule selects the lowest feasible threshold, τ* = 0.60; among the feasible candidates this is also the operating point with the largest communication reduction. At calibration, τ* yields scenario-balanced risk 0.517552 and a 14.02% communication reduction relative to *A*_5_. These calibration outcomes are used only to lock the operating point and do not constitute confirmatory statistical evidence.

(10) $$\tau^{*}=0.60$$

After the operating-point lock, the feature set, action order, action candidates, ensemble checkpoints, threshold, *α_cal_*, and non-inferiority margin cannot be modified. No threshold sweep or policy reselection is permitted after confirmatory access.

### 3.6. Operational Resource and Sustainability Accounting

EcoCert-V2X evaluates communication reduction together with computational and environmental consequences. Communication volume is determined exactly by Equations (2) and (3). The communication quantities reported here represent raw application-level semantic payloads rather than achievable throughput over a particular V2X protocol stack. For one collaborating peer at a 10 Hz message rate, the corresponding raw payload rates are 31.46 Mbps for *A*_1_, 125.83 Mbps for *A*_3_, and 503.32 Mbps for *A*_5_. These values do not include packetization, medium access control/physical layer (MAC/PHY) overhead, retransmissions, scheduling efficiency, congestion, or end-to-end delivery deadlines, and should therefore not be interpreted as measured C-V2X, NR-V2X, or IEEE 802.11bd link throughputs. All runtime-dependent measurements were acquired on an NVIDIA A100-SXM4-40GB GPU (NVIDIA Corporation, Santa Clara, CA, USA) using Python 3.11.15, PyTorch 2.6.0+cu126, and CUDA 12.6. GPU action energy was instrumented through the NVIDIA Management Library (NVML) total-energy counter, which was verified to be supported by the device. The same measurement path was used for EcoCert-V2X and the *A*_5_ reference.

GPU action energy is measured separately from communication energy. Let *e_i_^start^* and ${e_{i}}^{end}$ denote the hardware total energy counter readings in mJ immediately before and after the measured action execution. The non-negative GPU action energy is

(11) $$E_{GPU,i}=max\;[\frac{e_{end,i}-e_{start,i}}{1000},\;0]\;\left[J\right]$$

Execution latency is measured directly around the same action path,

(12) $$L_{i}=1000\;\left(t_{end,i}-t_{start,i}\right)\;\left[ms\right]$$

Communication energy was modeled separately from the measured GPU action energy. Before confirmatory evaluation, a three-level sensitivity model was frozen with communication energy coefficients of 17.763, 65.789, and 243.421 nJ/bit for the low, nominal, and high settings, respectively. The primary operational analysis used the prespecified nominal value *η_comm_* = 65.789 nJ/bit.

(13) $$E_{comm,i}=Q_{i}\;\eta_{comm}$$

The nominal operational energy quantity is then

(14) $$E_{op,i}=E_{GPU,i}+E_{comm,i}$$

Operational carbon was derived using a likewise frozen three-level electricity–carbon–intensity model of 140, 415, and 640 gCO_2_e/kWh for the low, nominal, and high settings, respectively. The primary analysis used the prespecified nominal value *γ* = 415 gCO_2_e/kWh.

(15) $$C_{i}=\frac{E_{op,i}}{3.6\times 10^{6}}\;\gamma \;[g{CO}_{2}e]$$

The disclosed accounting separates measured GPU action energy, modeled communication energy, and derived operational carbon. The reduction in transmitted data cannot be understood as equivalent to lowering GPU action energy. This difference is necessary for the subsequent hypothesis tests and is consistent with the Green AI principles of analyzing resource and environmental aspects explicitly [40,41].

### 3.7. Frozen Evaluation Principle

EcoCert-V2X follows a prospective development–calibration–confirmatory strategy. In this workflow, training and development data are used to construct the context representation and action-admissibility models, while calibration is used only to freeze the final operating threshold and the calibration-derived risk target. Once these elements are fixed, confirmatory evaluation is performed without retraining the detector or selector, modifying the feature set or threshold, changing the action set, or redesigning the system based on confirmatory outcomes. The same frozen policy is retained for the robustness analysis. This design makes it possible to distinguish absolute risk admissibility from relative perception preservation. Accordingly, a communication-efficient policy may remain non-inferior to full cooperation within the prespecified margin even if it does not satisfy the stricter absolute risk target. Communication efficiency, latency, energy-related quantities, and operational carbon are evaluated alongside these perception criteria rather than being optimized retrospectively after the confirmatory results are observed.

## 4. Experimental Setup and Evaluation Protocol

### 4.1. Dataset and Scenario-Level Partitioning

Experiments were conducted on V2V4Real, a vehicle-to-vehicle cooperative perception benchmark containing synchronized real-world LiDAR observations collected from multiple connected vehicles [6]. The benchmark provides heterogeneous traffic density, occlusion patterns, peer availability, and inter-vehicle geometry, making it suitable for evaluating adaptive communication decisions under realistic cooperative perception contexts.

To prevent information leakage across highly correlated frames, all partitions were defined at the scenario level rather than by randomly splitting individual samples. The development partition contained 752 samples from five scenarios and was used for feature construction, model validation, and action policy design. The action admissibility models were fitted using a separate training partition containing 5297 samples from 22 scenarios. A separate calibration partition was reserved for freezing the operating threshold and risk allowance. The confirmatory partition was not used for model selection, threshold selection, or action set revision.

The final confirmatory evaluation contained 1846 samples from eight previously isolated scenarios. All scenario identities, feature definitions, action mappings, model parameters, decision thresholds, and inferential rules were fixed before the confirmatory outcomes were inspected. The primary perception estimand was scenario-balanced, so that each of the eight confirmatory scenarios contributed equally to the final risk estimate irrespective of its number of frames or detected objects. The frozen experimental protocol is summarized in Table 3.

### 4.2. Cooperative Perception Backbone and Frozen Action Execution

The perception pipeline used the spatiotemporal cooperative perception backbone defined in the frozen implementation, with EcoCert-V2X operating as an adaptive cooperation controller above the detector. The controller changes the amount of peer information supplied to the perception backbone but does not modify the detector during confirmatory evaluation. The development action lattice comprised six predefined communication levels, whereas the final controller was frozen over *A*_0_, *A*_1_, *A*_3_, and *A*_5_, with *A*_5_ serving as the full-cooperation fallback.

The action budgets correspond to predefined fractions of the peer spatial feature map: *A*_0_ transmits no peer cells, *A*_1_ transmits 384 cells (6.25%), *A*_3_ transmits 1536 cells (25%), and *A*_5_ transmits 6144 cells (100%). Each spatial cell contributes 1024 bytes to the communication accounting. The frozen selector evaluates the candidate low-cost actions in the order *A*_0_ → *A*_1_ → *A*_3_ and selects the first action whose ensemble admissibility score reaches τ* = 0.60; if none satisfies the threshold, the system falls back to *A*_5_. The action selected for a sample is therefore determined before the corresponding detection outcome is evaluated.

### 4.3. Perception and Resource Metrics

Perception quality was quantified using the scenario-balanced distance-weighted miss risk defined in Section 3. Detection matching used the frozen three-dimensional intersection-over-union criterion IoU3D ≥ 0.70. For each scenario s, weighted missed ground-truth mass was normalized by the corresponding weighted ground-truth mass, and the primary risk was obtained by averaging scenario-specific risks with equal scenario weights.

(16) $$R_{s}\left(a\right)=\frac{\sum_{i}\;m_{si}\left(a\right)}{\sum_{i}\;g_{si}},\;R\left(a\right)=\frac{1}{S}\;\sum_{s}\;R_{s}\left(a\right)$$

Operational evaluation included communication volume, communication energy, GPU action energy, latency, and operational carbon. Communication volume was determined directly by the selected action and was converted to bits using the frozen action-specific peer-cell budget. Communication energy was modeled using the frozen coefficient *η_comm_* = 65.789 nJ/bit. GPU action energy was measured using the hardware total energy counter, whereas operational carbon was derived from total operational energy using the frozen carbon intensity factor *γ* = 415 gCO_2_e/kWh.

(17) $$E_{comm,i}=Q_{i}\;\cdot \;\eta_{comm},\;C_{i}=\frac{E_{GPU,i}+E_{comm,i}}{3.6\times 10^{6}}\;\cdot \;\gamma$$

For a resource quantity M, the percentage reduction relative to full cooperation was computed as

(18) $$\Delta_{M}=100\times [1-\frac{M_{EcoCert}}{M_{A5}}]$$

Communication volume and communication energy reduction are deterministic once the action sequence is fixed. Runtime-dependent quantities were evaluated over 30 paired operational seeds, with the same seed used for EcoCert-V2X and *A*_5_. Although the resource log contains many per-action observations, formal resource inference was conducted at the level of the paired operational seeds rather than treating individual runtime rows as independent replicates.

### 4.4. Calibration and Confirmatory Statistical Protocol

Calibration used a separate set of 1056 samples from five scenarios. Candidate thresholds were evaluated on the frozen grid {0.20, 0.30, 0.40, 0.50, 0.60, 0.70, 0.80, 0.90, 0.95}. The full-cooperation calibration risk was 0.543035. The prespecified margin δ=0.03 was defined as an operational tolerance on the scenario-balanced miss-risk scale rather than as a regulatory or safety-certified threshold. During calibration, this margin yielded the absolute target $\alpha_{cal}=0.573035$. The same margin was subsequently used as the relative non-inferiority tolerance with respect to the *A*_5_ full-cooperation reference. It represents an allowable absolute increase of 0.03 on the task-risk scale, and was fixed before any confirmatory outcome was accessed. The operating threshold was then locked at $\tau^{*}=0.60$ before confirmatory evaluation. 

Relative perception preservation was assessed through non-inferiority against *A*_5_. Let *Ds* = *Rs* (πτ*) − *Rs* (*A*_5_) denote the paired scenario-specific risk difference, where πτ* denotes the frozen EcoCert-V2X policy at τ* = 0.60. The principal relative criterion was

(19) $$\Delta_{R}=R\left(\pi_{\tau^{*}}\right)-R\left(A_{5}\right)<\delta ,\;\;\delta =0.03$$

The confirmatory analysis is based on classical scenario-level parametric inference rather than conformal inference; the reported confidence bounds and decisions therefore rely on the prespecified Student-t procedures described below.

Confirmatory inference was conducted at the prespecified aggregation level rather than at the individual frame level. For absolute risk admissibility (H1), a one-sample, one-sided Student’s *t*-test was applied to the eight scenario-specific EcoCert-V2X risk values against the calibration-derived target $\alpha_{cal}$; the corresponding one-sided 95% Student-t upper confidence bound was also reported. For relative perception non-inferiority (H4), a one-sample, one-sided Student’s *t*-test was applied to the eight paired scenario-level differences $D_{s}=R_{s}\left(\pi_{\tau^{*}}\right)-R_{s}\left(A_{5}\right)$, testing $H_{0}:E\left[D_{s}\right]\geq \delta$ against $H_{1}:E\left[D_{s}\right]<\delta$. Non-inferiority was established when the corresponding one-sided 95% Student-t upper confidence bound was below δ=0.03. Runtime-dependent resource effects were evaluated at the level of the 30 matched operational seeds, with EcoCert-V2X and *A*_5_ paired within each seed; individual resource-log rows were not treated as independent replicates. The composite sustainability hypothesis H3 followed an intersection–union decision rule requiring support for both its GPU action energy and operational-carbon components. Multiplicity across the prespecified primary stochastic hypothesis family was controlled using Holm’s step-down procedure. The primary confirmatory inference procedures and decision rules are summarized in Table 4.

### 4.5. Robustness Evaluation Under Communication Degradation

After the clean confirmatory evaluation, the same frozen policy was tested under six predefined communication perturbations without retraining, threshold modification, or policy reselection. The first perturbation family modeled random semantic-cell erasures in the communicated representation. ERASURE_10, ERASURE_20, and ERASURE_30 correspond to erasure rates of 10%, 20%, and 30%, respectively. The second family modeled additive white Gaussian noise (AWGN) on the communicated semantic representation. SNR_30, SNR_20, and SNR_10 correspond to target signal-to-noise ratios of 30 dB, 20 dB, and 10 dB, respectively, with lower SNR representing stronger communication degradation. Each perturbation condition was evaluated using three fixed perturbation seeds across all eight confirmatory scenarios, while the EcoCert-V2X selector, action set, threshold, and perception model remained unchanged.

Robustness was examined at two complementary resolutions. Condition-level analyses quantified the average and worst-seed relative risk gaps against the same non-inferiority margin *δ* = 0.03. A finer condition × scenario × seed decomposition was also retained to identify localized sensitivity that could be hidden by aggregate summaries. The local analysis therefore contained 6 × 8 × 3 = 144 perturbation evaluations.

### 4.6. Reproducibility and Experimental Governance

All confirmatory and robustness procedures were executed from versioned scripts and frozen configuration artifacts. No model retraining, feature redesign, threshold reselection, action lattice modification, or post-confirmatory tuning was permitted. Derived results, statistical outputs, figures, tables, status files, and integrity manifests were preserved with cryptographic hashes. This governance ensures that the results reported below correspond to the same fixed EcoCert-V2X policy defined before the confirmatory outcomes were inspected.

## 5. Results

### 5.1. Experimental Evidence Scale and Frozen Calibration

The final evaluation was built from several complementary evidence surfaces rather than from a single aggregate test. Table 5 summarizes the scale of the frozen calibration, confirmatory, operational, and robustness analyses. The 110,760 operational rows represent repeated paired measurements across 30 seeds and must not be interpreted as 110,760 independent statistical replicates; formal resource inference was conducted at the paired seed level. Likewise, the robustness surface was retained at both aggregate and local resolutions so that condition-level stability could be distinguished from scenario-specific sensitivity.

Calibration was performed on 1056 samples from five scenarios using the nine frozen candidate thresholds. Figure 2 shows the resulting risk-communication landscape. The calibration-derived absolute risk allowance was *α_cal_* = 0.573035, and the final operating threshold was locked at τ* = 0.60 before confirmatory evaluation. At this operating point, the calibration scenario-balanced task risk was 0.517552 and the communication reduction relative to *A*_5_ was 14.0211%. The recorded feasibility flags identify 0.60, 0.70, and 0.80 as feasible operating points; no threshold was reselected after the lock. The complete frozen calibration threshold sweep, including task risk, communication reduction, carbon reduction, and feasibility status, is reported in Table 6.

### 5.2. Confirmatory Perception Preservation and Adaptive Cooperation

The locked confirmatory evaluation contained 1846 samples from eight isolated scenarios. EcoCert-V2X obtained a scenario-balanced task risk of 0.651313, compared with 0.643265 for the full-cooperation reference *A*_5_. The paired scenario-balanced difference was therefore +0.008048. The one-sided 95% upper confidence bound was +0.018756, which remained below the prespecified non-inferiority margin *δ* = 0.03. The corresponding raw *p*-value was 0.003011 and the Holm-adjusted *p*-value was 0.009033. Relative perception non-inferiority was therefore formally supported for the prespecified scenario-balanced estimand. The margin-sensitivity analysis and the simple-policy baseline comparisons reported below are secondary post-hoc exploratory analyses conducted using the already frozen confirmatory artifacts. They were not part of the prespecified confirmatory hypothesis-testing protocol and do not alter the primary confirmatory decision. A sensitivity analysis was conducted across non-inferiority margins ranging from 0.01 to 0.05. With the one-sided 95% upper risk-gap bound fixed at +0.018756, non-inferiority was supported for tested margins of 0.020 and above, but not for 0.015 or 0.010. Thus, the confirmatory conclusion does not depend exclusively on the originally prespecified value of 0.03, although it would not hold under the stricter tested margins of 0.015 or 0.010. Importantly, this evidence establishes relative non-inferiority with respect to *A*_5_, and should not be interpreted as absolute risk admissibility, since the prespecified absolute-risk criterion was not satisfied in the confirmatory evaluation.

The individual paired risk differences for the eight confirmatory scenarios are reported in Supplementary Figure S1. These values provide a direct view of the scenario-level variability underlying the aggregate non-inferiority result. The scenario-specific differences are reported descriptively for transparency; the formal confirmatory decision remains based on the prespecified scenario-balanced paired analysis and its one-sided 95% Student-t upper confidence bound, rather than on requiring each individual scenario to satisfy the non-inferiority margin separately.

Figure 3 provides a sample-resolved view of this result. Confirmatory samples are ordered by *A*_5_ task risk and the curves report 35-sample moving means for each frozen cooperation action and for the EcoCert-V2X selector. The fixed actions separate most clearly over lower- and intermediate-risk regions, while the adaptive EcoCert-V2X trajectory tracks the high-cooperation profiles over much of the ordered surface. This visualization is descriptive; the formal non-inferiority decision remains based on the scenario-balanced paired analysis rather than on individual sample ranks.

The adaptive policy was conservative in its use of reduced cooperation. Across all 1846 confirmatory samples, *A*_0_ was selected 146 times, *A*_1_ 20 times, *A*_3_ 148 times, and *A*_5_ 1532 times, corresponding to approximately 7.9%, 1.1%, 8.0%, and 83.0% of samples, respectively. The predominance of *A*_5_ reflects the conservative fallback design of the controller rather than an objective of maximizing communication compression. Figure 4 shows that this mixture was strongly scenario-dependent. S2, S3, and S5 used *A*_5_ for all confirmatory observations, S4 and S7 were also dominated by *A*_5_, whereas S0, S1, and S6 contained much larger shares of reduced-cooperation actions. The selector therefore did not impose a uniform compression level across the confirmatory set.

The resulting action sequence reduced total transmitted communication by 14.9377% relative to always using *A*_5_. Because communication energy was modeled linearly from transmitted bits, the communication energy reduction was also 14.9377%. Scenario-wise effects were heterogeneous, so the confirmatory conclusion is deliberately stated at the prespecified scenario-balanced level, rather than as a claim that every scenario individually satisfies the same relative risk bound. The comparison with representative simple cooperation policies on the frozen confirmatory action surface is summarized in Table 7.

To provide additional comparative context, the frozen confirmatory action surface was evaluated against representative simple cooperation policies without retraining or modifying EcoCert-V2X. Fixed *A*_1_ and *A*_3_ achieved communication reductions of 93.75% and 75.00%, respectively, while preserving aggregate non-inferiority at δ=0.03. In particular, fixed *A*_3_ obtained a lower aggregate task risk than EcoCert-V2X, indicating that the adaptive controller does not dominate all fixed-budget policies on this benchmark. However, under 1000 scenario- and peer-count-matched random allocations with the same 14.94% communication reduction as EcoCert-V2X, the mean risk was 0.655705, and only 0.2% of the random allocations achieved a risk no greater than EcoCert-V2X. These results suggest that the context-dependent placement of reduced-cooperation actions is more informative than matched random allocation, while fixed-budget policies may provide a stronger aggregate risk–communication trade-off in some operating regimes. These comparisons are intended as controlled policy baselines evaluated on the same frozen confirmatory action surface, and should not be interpreted as a head-to-head benchmark against reimplemented state-of-the-art communication-efficient cooperative-perception methods.

### 5.3. Operational Efficiency and Carbon Stability Across Paired Seeds

The operational analysis combined the deterministic communication effect with 30 paired runtime seeds for GPU action energy, latency, and operational carbon. Figure 5 presents a cumulative paired-seed ratio-of-sums profile over the 1846 confirmatory samples ordered by *A*_5_ task risk. To avoid unstable ratios when the cumulative reference denominator is extremely small, the display begins after a 35-sample warm-up; all 1846 samples nevertheless remain included in the final cumulative estimates. The communication curve converges to a 14.94% reduction and the operational carbon curve to a 4.07% reduction. The operational reductions across the 30 paired seeds are summarized in Table 8.

Operational carbon decreased by 4.0678% on average, with a 95% confidence interval of 3.3390% to 4.7966% and positive aggregate reductions in 29 of the 30 paired seeds. Figure 6 resolves this effect into 240 scenario-by-seed cells. The strongest reductions occur in a subset of scenarios, particularly S0 and S1, while several scenarios show small or locally negative values for some seeds. The supported carbon result is therefore an aggregate operational effect rather than a claim of a positive reduction for every scenario–seed pair.

The GPU action energy reduction was much smaller—0.4465% on average, with a 95% confidence interval from −0.5275% to 1.4206%—and only 18 of 30 seeds yielded a positive reduction. Latency decreased by 0.0927% on average. Consequently, the principal operational evidence is the deterministic reduction in communication and the supported reduction in operational carbon; the GPU action energy result is treated as unconfirmed rather than generalized as an energy efficiency gain.

### 5.4. Robustness Under Frozen Communication Degradation

Robustness was evaluated without retraining, threshold modification, or policy mutation under three semantic cell erasure settings and three semantic AWGN settings, each with three fixed perturbation seeds. At the condition level, all six settings preserved the aggregate relative non-inferiority criterion. Mean condition-level risk gaps ranged from −0.006853 under ERASURE_30 to +0.008377 under SNR_20, and the corresponding worst seed gaps also remained well below *δ* = 0.03. The condition-level robustness results for the six frozen communication perturbations are summarized in Table 9. Figure 7 further illustrates the mean and worst-seed task-risk gaps across these six perturbation conditions relative to the prespecified non-inferiority margin.

The condition averages do not imply uniform behavior at finer resolutions. Figure 8 decomposes robustness by condition and scenario. The largest positive mean gaps are concentrated in S0 and S1 under SNR perturbation, while many cells remain at or near zero, and several favor EcoCert-V2X. Across the 48 condition-by-scenario combinations, 42 preserved the all-seed non-inferiority criterion. At the finest condition-by-scenario-by-seed resolution, 13 of 144 cells exceeded the margin, corresponding to 9.03% of the local evaluations; the worst local gap was +0.040840. These departures were concentrated in the same S0-S1 region, especially under SNR degradation.

Taken together, the robustness results support aggregate stability across the six prespecified degraded-communication conditions while also defining a clear limitation: relative performance can deteriorate locally for specific scenario–condition combinations. The evidence therefore supports robustness at the prespecified aggregate level, not an unconditional guarantee across all scenarios, seeds, and channel perturbations.

### 5.5. Multiplicity-Controlled Confirmatory Evidence

Table 10 consolidates the prespecified confirmatory decisions. Relative perception non-inferiority (H4) remained supported after Holm correction, with an adjusted *p*-value of 0.009033 and a one-sided 95% upper risk-gap bound of +0.018756 below *δ* = 0.03. The communication reduction is a deterministic resource result from the frozen action sequence. The operational carbon submetric was also supported, with a mean reduction of 4.067817% and a 95% confidence interval of 3.3390% to 4.7966%.

The absolute-risk admissibility target H1 was not supported; the confirmatory risk was 0.651313 and its one-sided 95% upper bound was 0.693894. This result is distinct from the supported relative non-inferiority statement. The composite sustainability hypothesis H3 was also not supported because the GPU action energy component was not independently confirmed, even though the operational carbon submetric was strongly supported. These distinctions preserve the frozen claim boundaries and avoid conflating relative perception preservation, deterministic communication savings, and runtime-dependent energy effects.

## 6. Discussion

The results indicate that EcoCert-V2X can reduce cooperative perception resource demand while maintaining task performance within a predefined relative risk tolerance. At the frozen operating point, the framework reduced communication volume by 14.94% relative to full cooperation (*A*_5_), while the scenario-balanced task risk gap remained +0.0080 and its one-sided 95% upper confidence bound (+0.0188) stayed below the predefined non-inferiority margin of 0.03. This result is important because the communication saving was obtained through adaptive selection among *A*_0_, *A*_1_, *A*_3_, and *A*_5_, rather than through a uniform reduction of transmitted information. The scenario-wise action composition further shows that EcoCert-V2X preserves full cooperation when lower-cost actions are not sufficiently supported by the frozen selector, while exploiting substantial communication reductions in scenarios where reduced cooperation remains appropriate.

The additional baseline analysis also shows that EcoCert-V2X should not be interpreted as maximizing communication reduction on this benchmark. Its contribution lies instead in context-dependent, risk-aware cooperation selection under a frozen decision protocol, rather than in universal dominance over fixed-budget policies. Accordingly, the present results do not establish superiority over state-of-the-art communication-efficient cooperative perception methods, whose fair comparison would require method-specific retraining and evaluation under a common backbone, partitioning protocol, and communication accounting framework.

The sustainability results also reveal a meaningful distinction between deterministic communication savings and hardware-dependent operational effects. The reduction in communication volume was fixed by the selected action sequence and therefore remained identical across the 30 operational seeds. In contrast, operational carbon depended partly on measured computation behavior and consequently exhibited greater seed-level variability. Nevertheless, the mean operational carbon reduction reached 4.07%, with positive reductions in 29 of 30 seeds, and this submetric was statistically supported. The cumulative and scenario–seed analyses further indicate that this benefit is not attributable to a single isolated execution or scenario. By comparison, the measured GPU action energy reduction was small and was not formally supported, suggesting that communication-aware cooperation does not automatically translate into a measurable reduction in every hardware energy component. Accordingly, the composite sustainability hypothesis H3 was not supported, despite the statistically supported operational carbon submetric.

The robustness tests provide further evidence that the frozen policy is not just effective in nominal communication conditions. The results show that non-inferiority remains in all six tested perturbation conditions for packet erasures and a weaker signal-to-noise ratio. However, the condition- and scenario-level analyses reveal localized departures from the aggregate trend, particularly for scenarios S0 and S1 under some SNR conditions. Thus, EcoCert-V2X exhibits robustness at the aggregate level, but not uniformly across all local scenario–condition combinations.

Several limitations should be considered when interpreting these results. While the existence of relative non-inferiority regarding *A*_5_ was formally supported, the calibration-derived absolute risk target was not reached. A further limitation is that the primary task risk metric does not directly incorporate false-positive detections. Future extensions should therefore complement the frozen miss risk estimand with false-positive-sensitive measures such as precision or a joint false-positive/false-negative risk. Moreover, the sustainability assessment is focused on operational effects of communication and computation, and should not be interpreted as a complete life-cycle environmental analysis. Future studies may cover EcoCert-V2X development related to more cooperative perception datasets, other communication conditions, and various types of hardware, as well as methods for adaptation, making sure that the distinction between calibration and independent evaluation is maintained.

## 7. Conclusions

This study introduced EcoCert-V2X, a risk-aware artificial intelligence framework for adaptive cooperative perception in connected-vehicle environments. The proposed approach selects among predefined cooperation levels using a frozen admissibility rule, allowing communication resources to be adjusted according to the operating context while retaining full cooperation as a fallback. The framework was assessed through a strictly separated development, calibration, and confirmatory protocol, with all decision rules and statistical criteria fixed before confirmatory evaluation.

At the frozen operating point, EcoCert-V2X reduced communication volume by 14.94% relative to the *A*_5_ full-cooperation reference. The scenario-balanced task-risk gap was +0.0080, with a one-sided 95% upper confidence bound of +0.0188, remaining below the prespecified non-inferiority margin of 0.03. This supports relative non-inferiority to *A*_5_, although the separate absolute risk target was not achieved. The operational carbon reduction reached 4.07% and was statistically supported, whereas the measured GPU action energy reduction was not formally confirmed. Accordingly, the composite sustainability hypothesis H3 was not supported. Additional baseline analysis further showed that EcoCert-V2X should not be interpreted as universally outperforming fixed-budget communication strategies; rather, its contribution lies in context-dependent, risk-aware allocation under a predefined decision protocol.

Robustness analysis showed that aggregate non-inferiority was maintained under all six tested packet erasure and SNR perturbation conditions, although localized sensitivity remained for specific scenario–condition combinations. These findings support adaptive cooperation as a means of reducing communication demand and operational carbon while maintaining perception performance within a predefined relative tolerance, but they do not establish universal robustness or protocol-level V2X feasibility. Future work will therefore extend the evaluation to broader datasets, heterogeneous sensing and hardware platforms, and complete V2X communication environments incorporating scheduling, congestion, retransmissions, and delivery constraints, while preserving strict separation between calibration, deployment decisions, and independent evaluation.

## Figures and Tables

**Figure 1:**
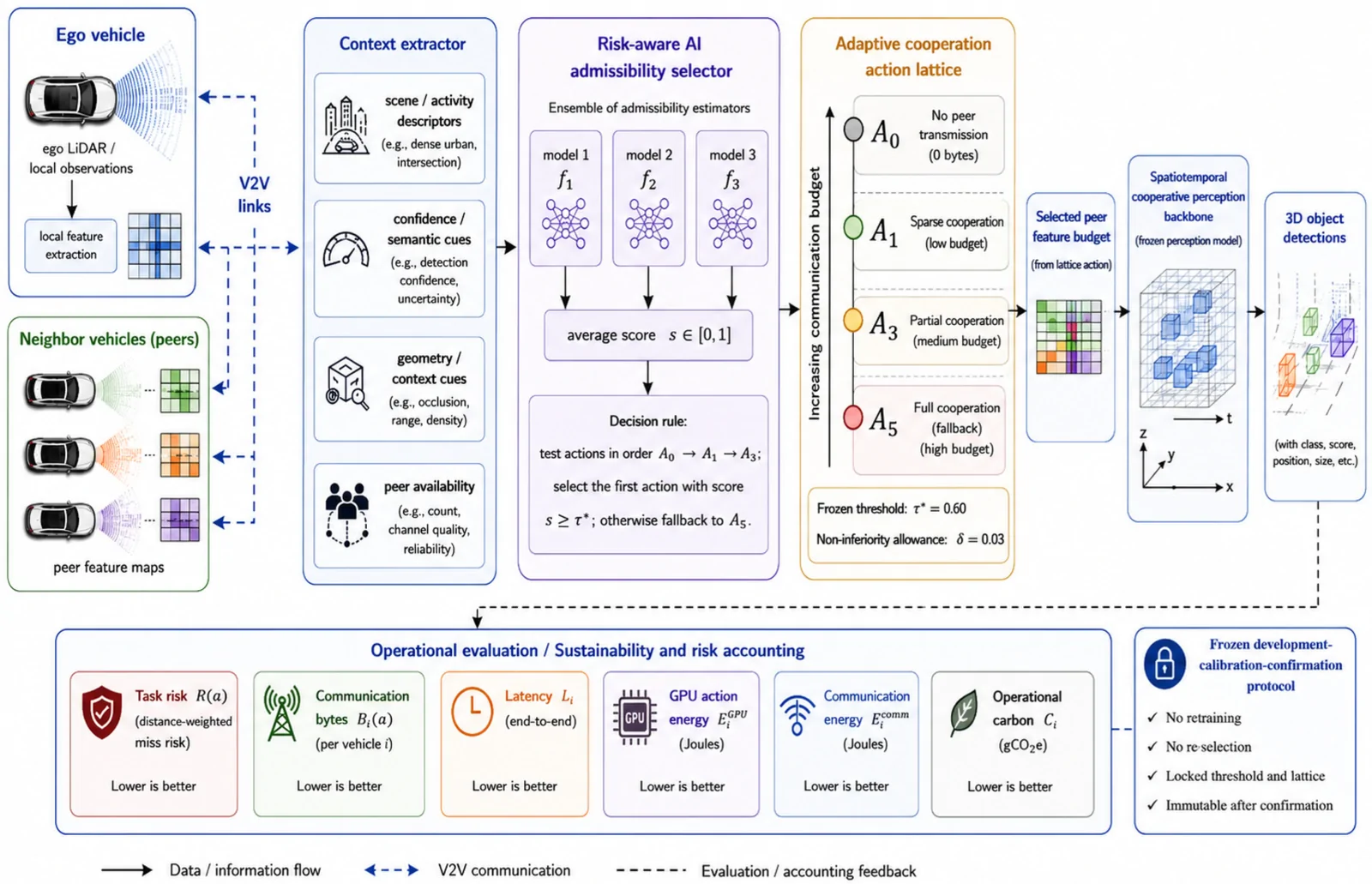
Overall EcoCert-V2X architecture. The frozen context and action-admissibility layer selects a predefined V2X cooperation budget before collaborative fusion; perception risk and operational resource accounting are evaluated through separate measurement paths.

**Figure 2:**
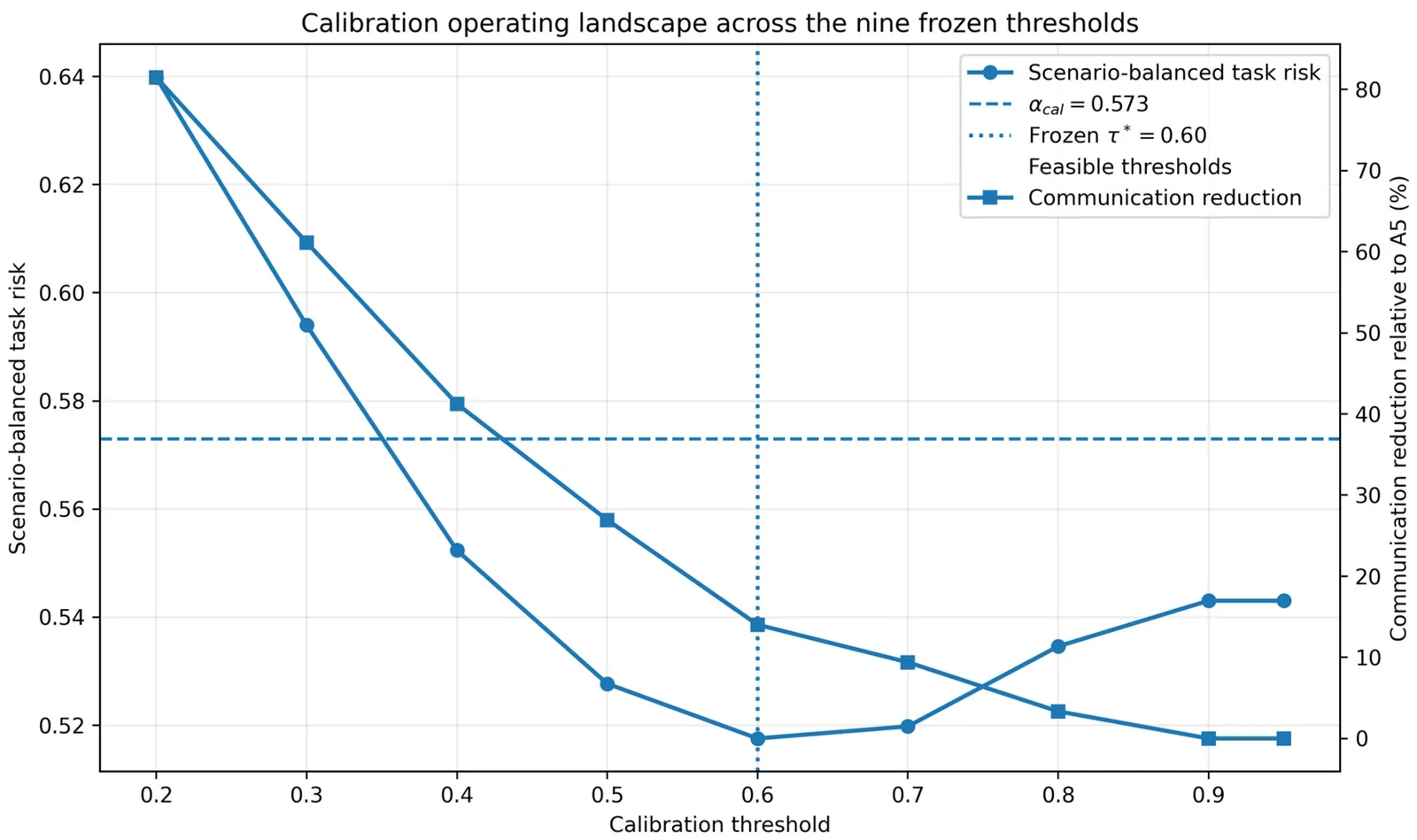
Calibration operating landscape across the nine frozen thresholds. The left axis reports scenario-balanced task risk, the right axis reports communication reduction relative to *A*_5_, the dashed horizontal line marks *α_cal_* = 0.573035, and the dotted vertical line marks the frozen threshold τ* = 0.60. This panel summarizes calibration only; no post-confirmatory threshold reselection was performed.

**Figure 3:**
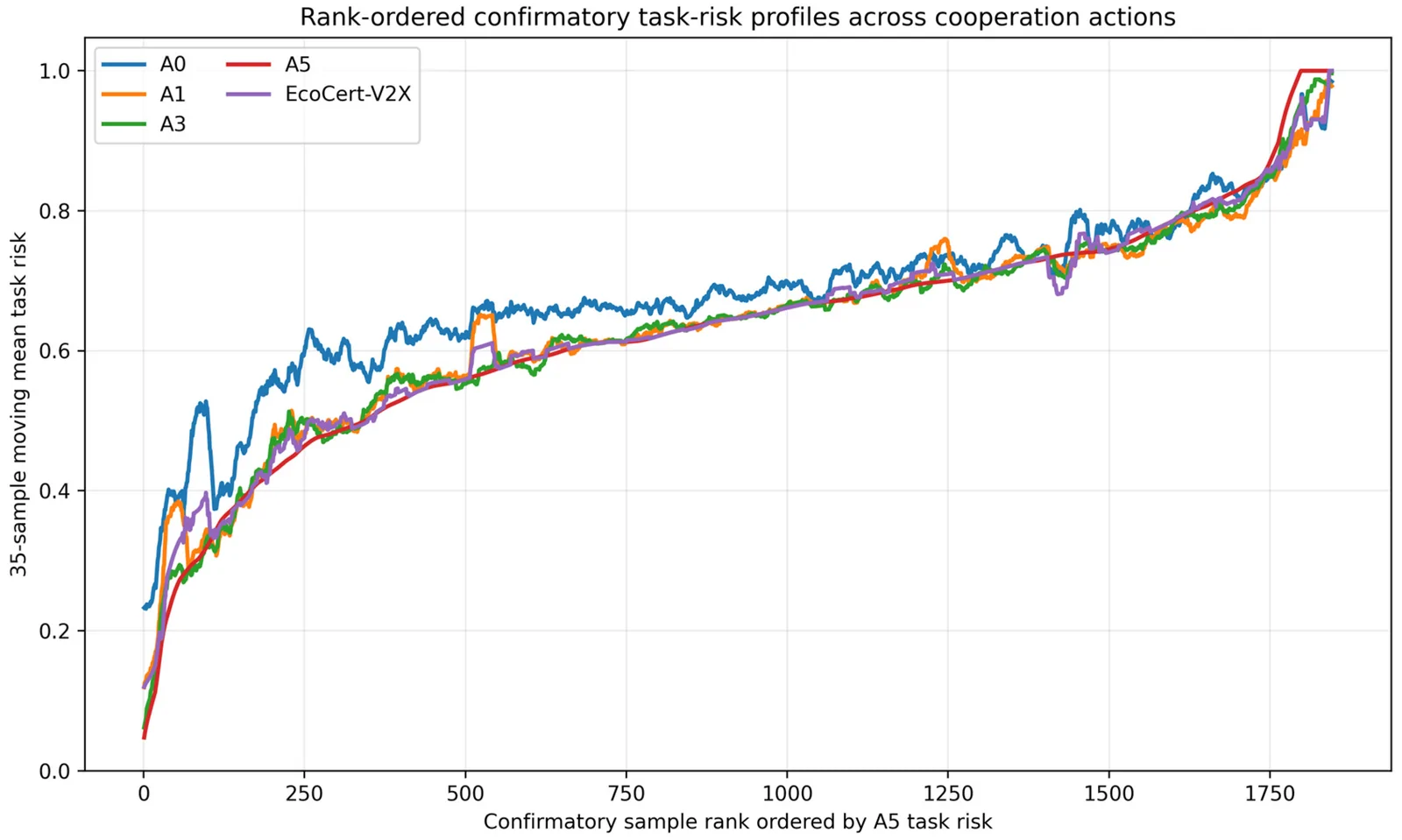
Rank-ordered confirmatory task-risk profiles across the frozen cooperation actions. Samples are ordered by *A*_5_ task risk and each curve is a 35-sample moving mean. The EcoCert-V2X curve corresponds to the frozen adaptive selector. The profile is descriptive and does not replace the scenario-balanced confirmatory inference.

**Figure 4:**
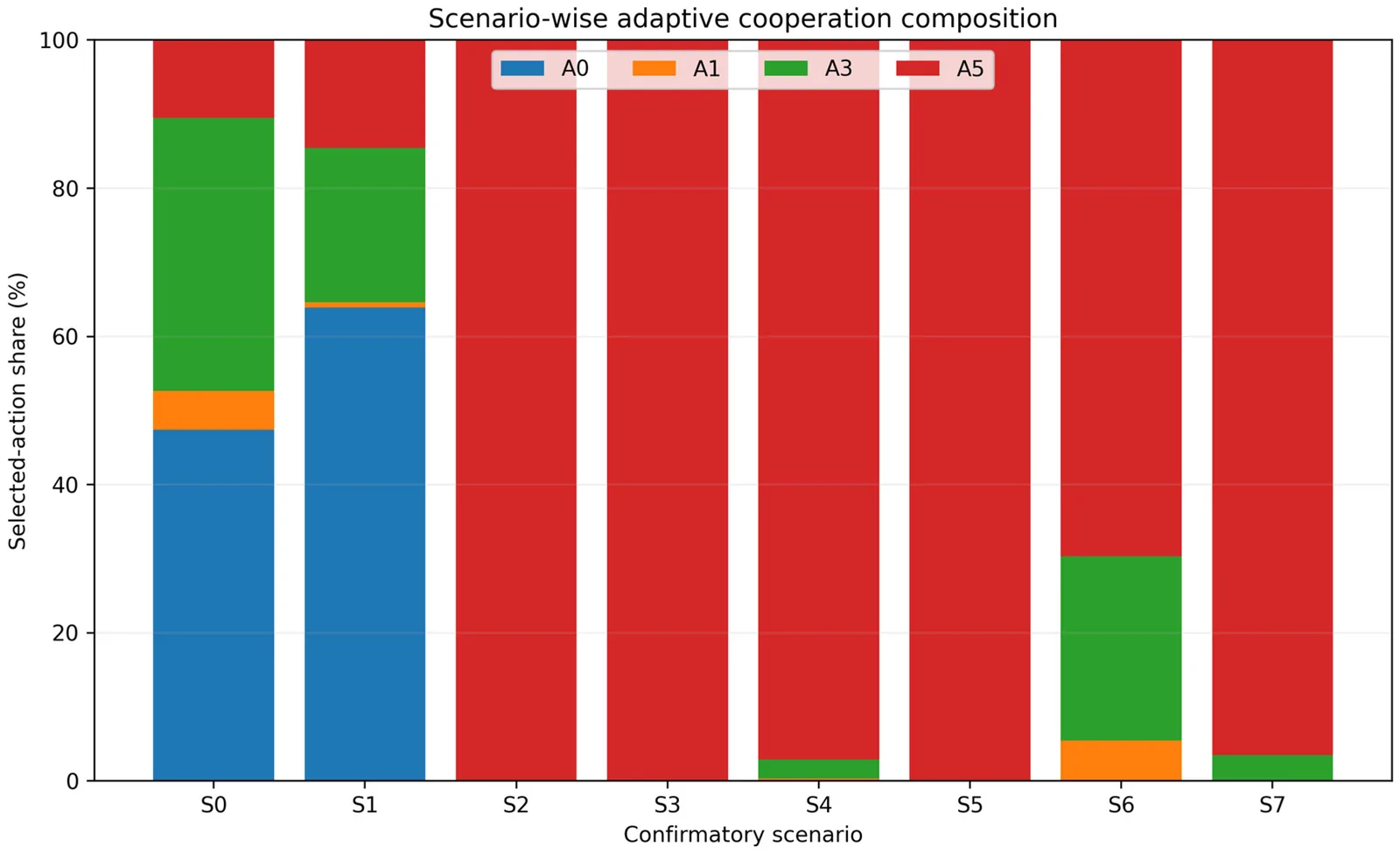
Scenario-wise composition of the frozen adaptive cooperation policy. Bars report the percentage of selected actions *A*_0_, *A*_1_, *A*_3_, and *A*_5_ within each confirmatory scenario. The distribution highlights the context-dependent use of reduced cooperation and the dominant full-cooperation fallback outside the scenarios in which lower-cost actions were frequently admitted.

**Figure 5:**
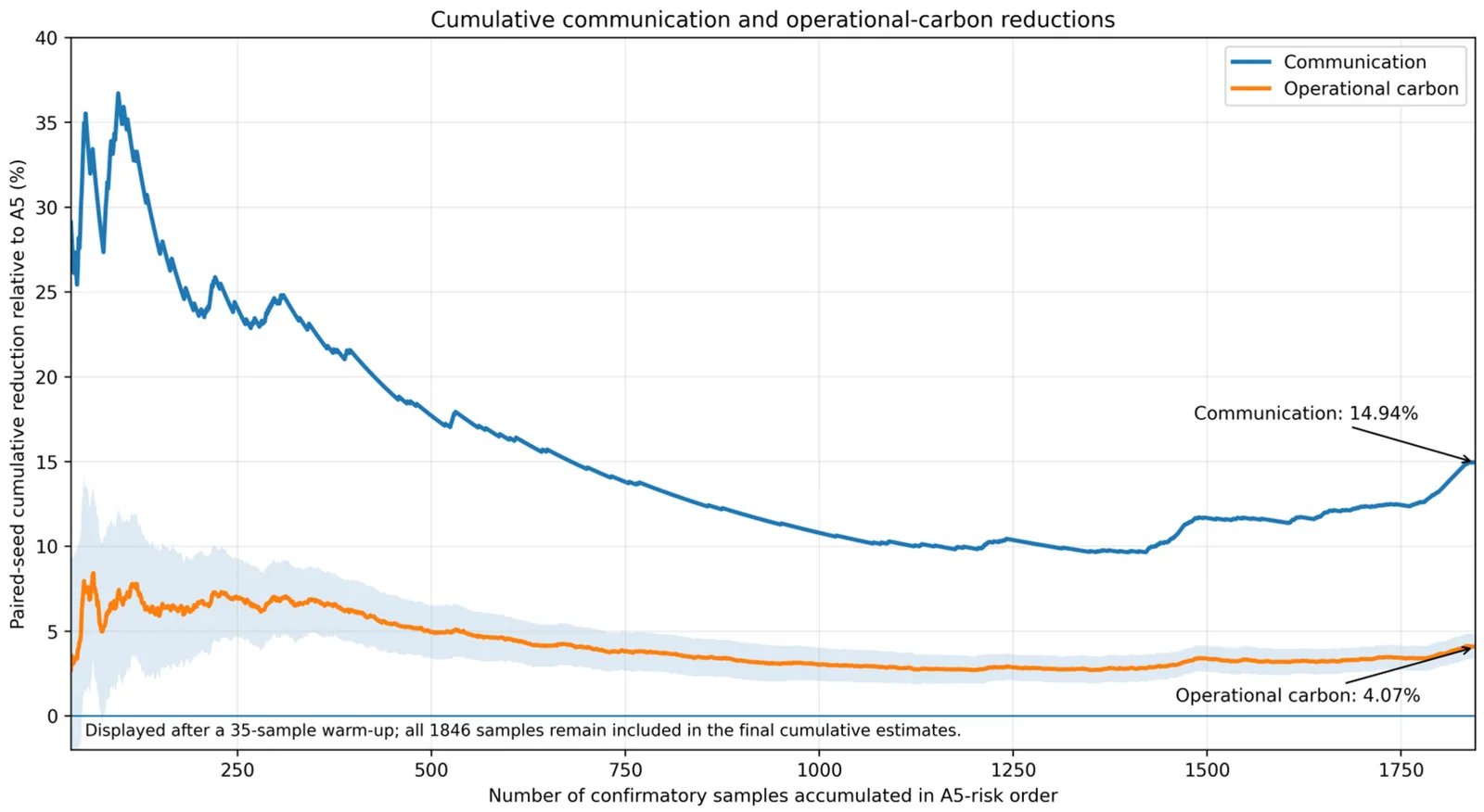
Cumulative communication and operational carbon reductions relative to *A*_5_. Curves are computed as paired-seed ratio-of-sums reductions along the *A*_5_-risk ordering. The display starts after a 35-sample warm-up to avoid unstable small-denominator ratios, while all 1846 samples contribute to the final cumulative estimates. The carbon band summarizes 95% uncertainty across the 30 paired operational seeds.

**Figure 6:**
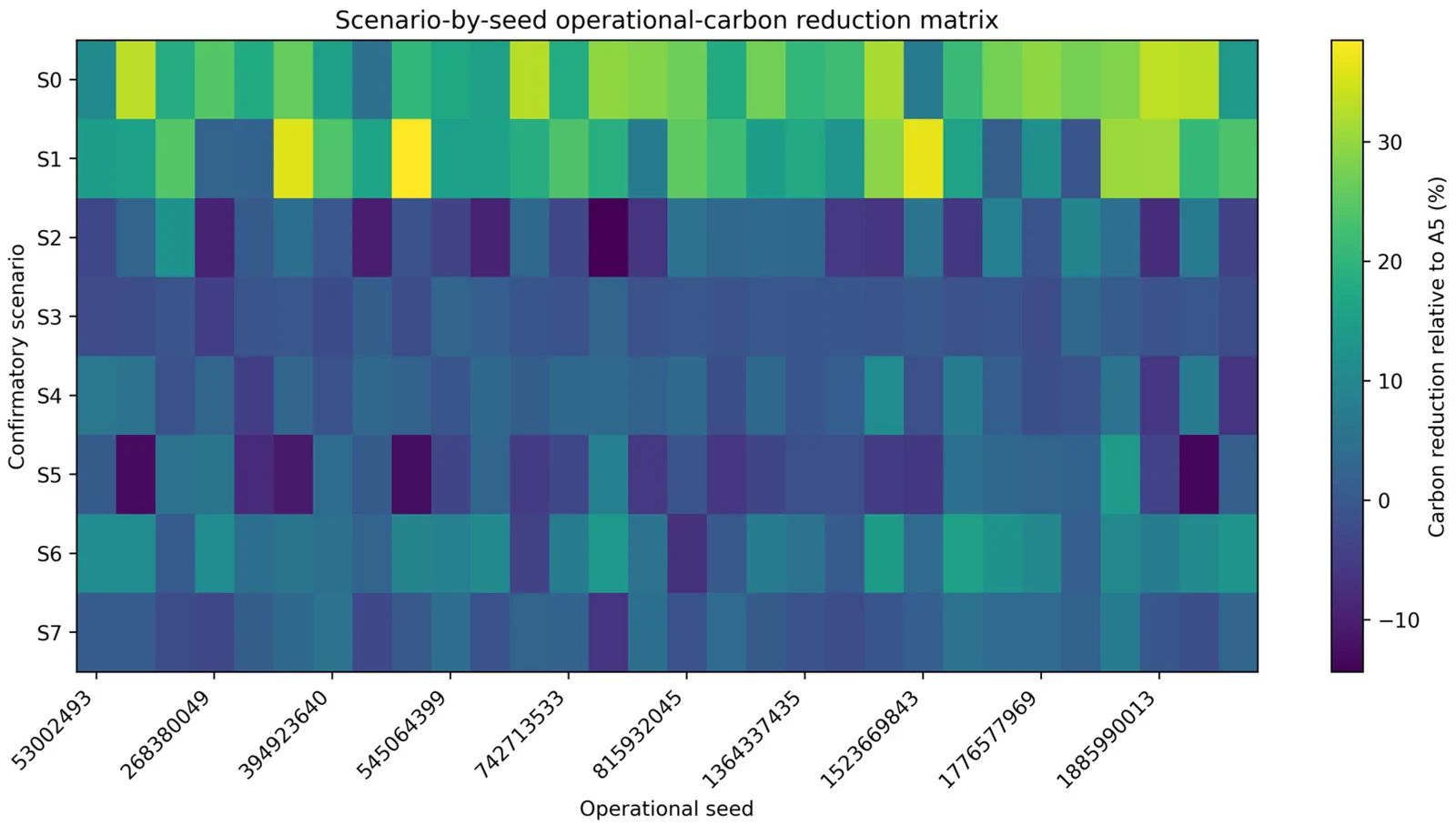
Scenario-by-seed operational carbon reduction matrix. Each cell reports the percentage reduction of EcoCert-V2X relative to *A*_5_ for one confirmatory scenario and one of the 30 paired operational seeds. The matrix exposes heterogeneity that is not visible in the aggregate mean.

**Figure 7:**
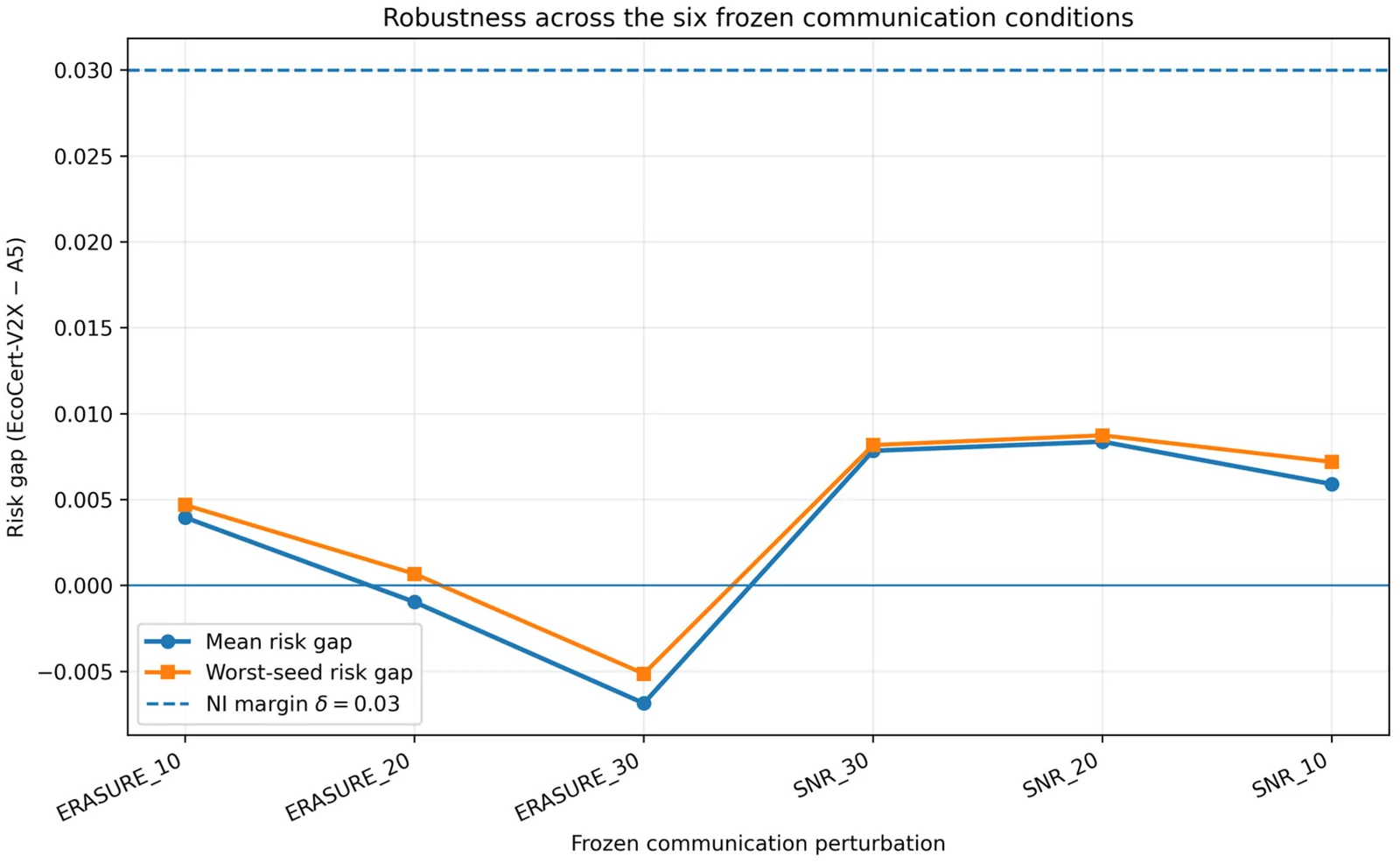
Condition-level robustness across the six frozen communication perturbations. Curves show the mean and worst seed task-risk gaps EcoCert-V2X minus *A*_5_. The dashed line marks the non-inferiority margin *δ* = 0.03. All six condition-level aggregate evaluations remain below the margin.

**Figure 8:**
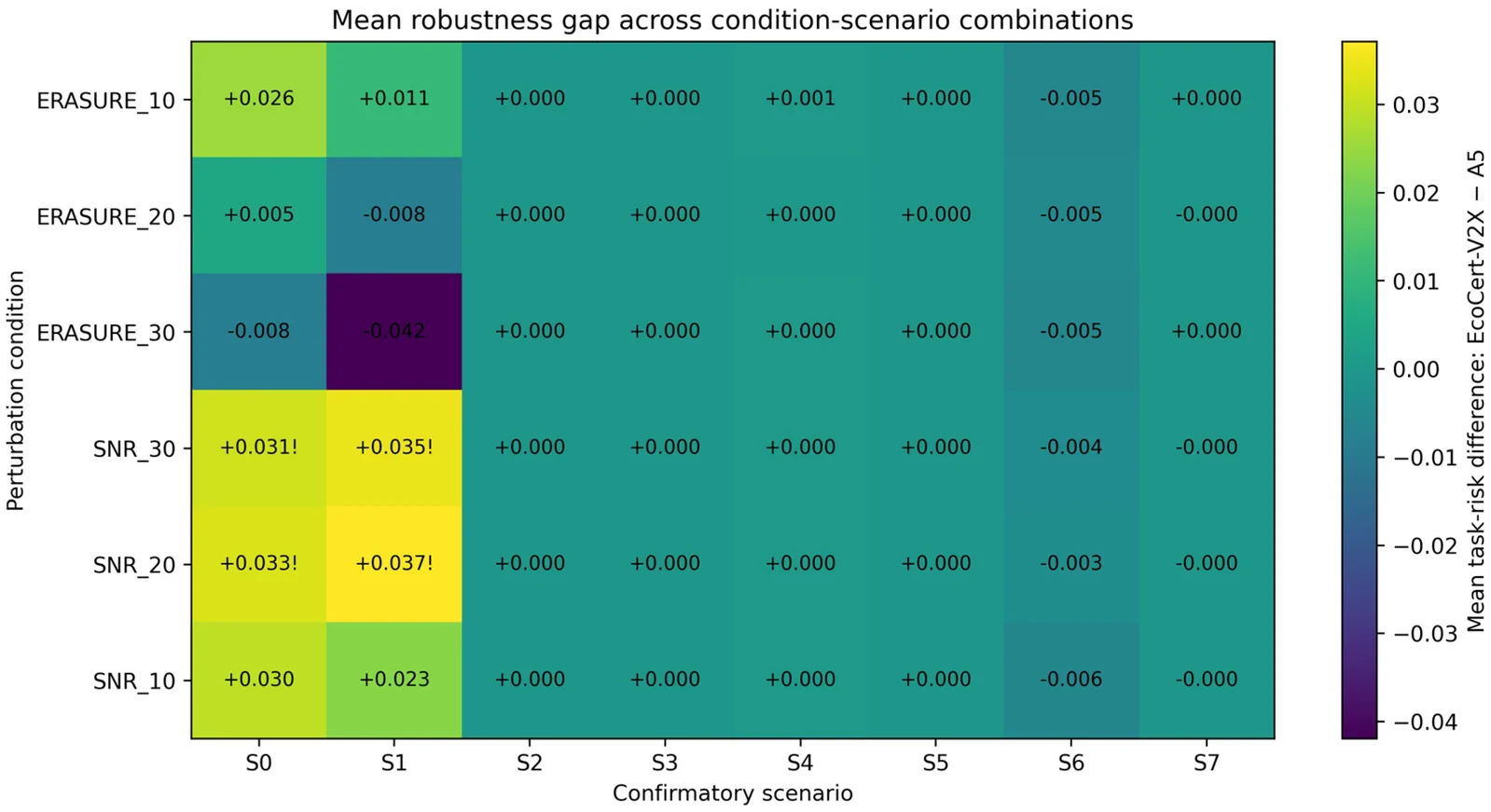
Mean condition-by-scenario robustness matrix. Each cell reports the mean task risk difference EcoCert-V2X minus *A*_5_ over the three perturbation seeds. Cells marked with an exclamation point have a mean gap greater than *δ* = 0.03. The matrix localizes sensitivity that is hidden by condition-level aggregation; it should not be interpreted as a claim of universal robustness.

**Table 1:** EcoCert-V2X cooperative action lattice and the role of the final controller.

Action	Peer Spatial Budget	Spatial Cells	Approx. Payload/Peer	Final Controller
*A*_0_	0%	0	0	Candidate
*A*_1_	6.25%	384	384 KiB	Candidate
*A*_2_	12.5%	768	768 KiB	Development only
*A*_3_	25%	1536	1536 KiB	Candidate
*A*_4_	50%	3072	3072 KiB	Development only
*A*_5_	100%	6144	6144 KiB	Fallback

**Table 2:** Development performance of the action-admissibility classifiers retained in the frozen operational selector.

Action	AUROC	Average Precision
*A*_0_	0.582	0.492
*A*_1_	0.656	0.689
*A*_3_	0.697	0.746

**Table 3:** Frozen experimental protocol.

Component	Frozen Setting
Dataset	V2V4Real [6]
Sensing modality	Cooperative LiDAR
Partition unit	Driving scenario
Confirmatory scenarios	8
Confirmatory samples	1846
Operational confirmatory seeds	30 paired seeds
Full-cooperation reference	*A*_5_
Frozen controller actions	*A*_0_, *A*_1_, *A*_3_, *A*_5_
Selector threshold	τ* = 0.60
Non-inferiority margin	*δ* = 0.03
Calibration-derived absolute target	*α_cal_* = 0.573035
Post-confirmatory retraining/reselection	Not permitted

**Table 4:** Primary confirmatory inference structure.

Question	Unit of Inference	Statistical Procedure/Decision Rule
Absolute task-risk admissibility (H1)	8 confirmatory scenarios	One-sample one-sided Student’s *t*-test against $\alpha_{cal}=0.573035$; one-sided 95% upper Student-t bound
Perception non-inferiority (H4)	8 paired scenarios	One-sample one-sided Student’s *t*-test on $D_{s}$; one-sided 95% upper Student-t bound <δ=0.03
Communication efficiency (H2)	Frozen selected actions	Deterministic reduction relative to *A*_5_; no stochastic test
GPU action energy effect	30 paired operational seeds	Paired seed-level one-sided inference versus *A*_5_
Operational-carbon effect	30 paired operational seeds	Paired seed-level one-sided inference versus *A*_5_
Composite sustainability (H3)	Two prespecified component tests	Intersection–union decision
Primary multiplicity control	Prespecified stochastic hypothesis family	Holm step-down adjustment

**Table 5:** Scale of the frozen experimental evidence used in the final analyses.

Evidence Block	Count	Interpretation
Calibration action measurements	4224	1056 samples × 4 actions
Confirmatory deterministic action surface	7384	1846 samples × 4 frozen actions
Confirmatory operational resource rows	110,760	1846 samples × 30 seeds × 2 roles
Confirmatory paired operational seeds	30	Paired EcoCert-V2X/*A*_5_ execution
Robustness logical result rows	66,456	1846 samples × 6 conditions × 3 perturbation seeds × 2 policies
Local robustness cells	144	6 conditions × 8 scenarios × 3 seeds

**Table 6:** Frozen calibration threshold sweep.

Threshold	Task Risk	Communication Reduction (%)	Carbon Reduction (%)	Feasible
0.20	0.639852	81.49	−10.75	No
0.30	0.594010	61.14	−7.64	No
0.40	0.552353	41.24	−4.87	No
0.50	0.527676	26.94	−4.26	No
0.60	0.517552	14.02	0.63	Yes
0.70	0.519790	9.39	1.99	Yes
0.80	0.534601	3.34	0.02	Yes
0.90	0.543035	0.00	0.00	No
0.95	0.543035	0.00	0.00	No

**Table 7:** Comparison with representative simple cooperation policies on the frozen confirmatory action surface.

Policy	Scenario-Balanced Task Risk	Communication Reduction vs. *A*_5_ (%)	One-Sided 95% Upper Risk Gap	NI at *δ* = 0.03
EcoCert-V2X	0.651313	14.94	0.018756	Yes
Local only (*A*_0_)	0.693480	100.00	0.070536	No
Full cooperation (*A*_5_)	0.643265	0.00	0.000000	Reference
Fixed *A*_1_ (6.25%)	0.651481	93.75	0.018559	Yes
Fixed *A*_3_ (25%)	0.648564	75.00	0.015378	Yes

**Table 8:** Operational reductions across the 30 paired seeds.

Metric	Mean (%)	Median (%)	SD (%)	Seed Range (%)	Positive Seeds
Communication volume	14.9377	14.9377	0.0000	[14.9377, 14.9377]	30/30
Communication energy	14.9377	14.9377	0.0000	[14.9377, 14.9377]	30/30
Operational carbon	4.0678	3.9800	1.9517	[−0.0149, 10.9034]	29/30
GPU action energy	0.4465	0.3552	2.6087	[−5.0684, 9.6659]	18/30
Latency	0.0927	0.0794	0.0561	[0.0008, 0.2389]	30/30

**Table 9:** Condition-level robustness of the frozen EcoCert-V2X selector.

Condition	EcoCert Risk	*A*_5_ Risk	Mean Gap	Worst Seed Gap	Aggregate NI
ERASURE_10	0.658642	0.654681	+0.003961	+0.004689	Yes
ERASURE_20	0.665696	0.666653	−0.000957	+0.000683	Yes
ERASURE_30	0.675325	0.682178	−0.006853	−0.005130	Yes
SNR_30	0.651481	0.643636	+0.007845	+0.008178	Yes
SNR_20	0.654040	0.645663	+0.008377	+0.008743	Yes
SNR_10	0.662735	0.656827	+0.005908	+0.007197	Yes

**Table 10:** Formal confirmatory evidence and multiplicity-controlled decisions.

Evidence	Estimand	Estimate	Bound/Interval	*p*-Value	Decision
H1	Absolute risk admissibility	risk = 0.651313	one-sided 95% upper = 0.693894	raw 0.994887; Holm 0.994887	Not supported
H2	Communication efficiency	communication reduction = 14.9377%	deterministic resource reduction; absolute admissibility premise not met	deterministic	Resource reduction supported
H3	Composite sustainability efficiency	GPU action energy = 0.446549%; operational carbon = 4.067817%	intersection union decision	raw 0.165763; Holm 0.331526	Not supported
H4	Relative perception non-inferiority	risk gap = +0.008048	one-sided 95% upper = +0.018756 < +0.030	raw 0.00301116; Holm 0.00903349	Supported
Carbon submetric	Operational carbon reduction	4.067817%	95% CI = [3.3390%, 4.7966%]	raw 4.43 × 10^−12^	Supported submetric
GPU submetric	GPU action energy reduction	0.446549%	95% CI = [−0.5275%, 1.4206%]	raw 0.165763	Not formally supported

## Data Availability

The technical resources supporting this study are available in the EcoCert-V2X project repository at [https://drive.google.com/drive/folders/1a2SbK92kIBBHvvQNenLCjywlTf8LxH_h?usp=sharing. accessed on 19 September 2026]. The shared reproducibility package includes the available source code, frozen experimental configurations and statistical contracts, calibration and confirmatory evaluation settings, derived evaluation results, robustness analyses, operational resource measurements, statistical outputs, technical tables, provenance records, and SHA-256 integrity manifests.

## Supplementary Materials

The following supporting information can be downloaded at: https://www.mdpi.com/article/10.3390/s26196025/s1, Figure S1: Scenario-level paired task-risk differences between EcoCert-V2X and *A_5_* across the eight confirmatory scenarios.
