# Supplementary material for: EcoCert-V2X: Risk-Aware Adaptive Cooperation for Communication-Efficient V2X Cooperative Perception
Source: Sensors (Basel). 2026 Sep 23;26(19):6025. doi: 10.3390/s26196025 (PMC13667674; doi:10.3390/s26196025)
Supplement: Supplementary file 1 [file sensors-26-06025-s001.zip › sensors-4557811-supplementary.pdf]

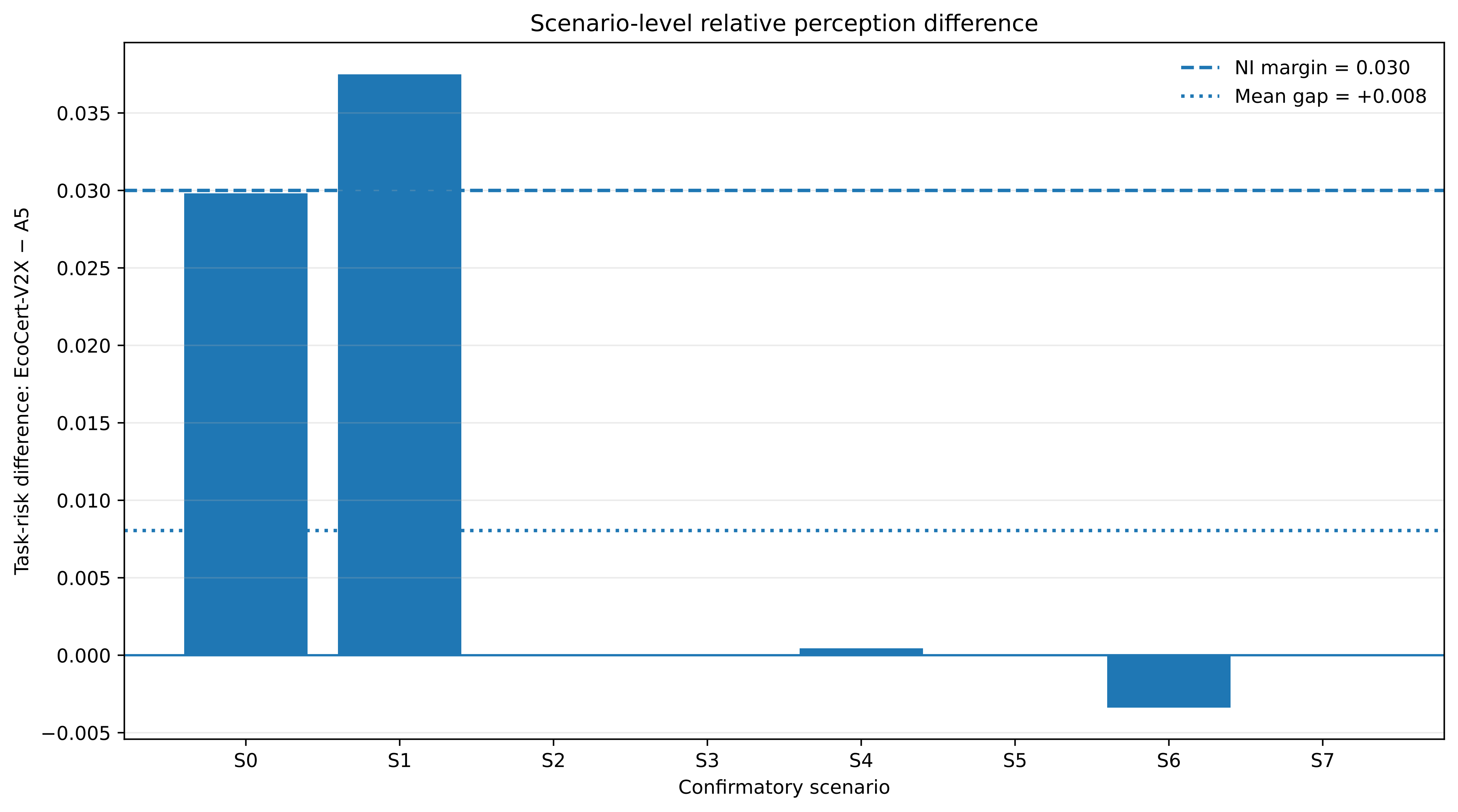

Scenario-level relative perception difference
NI margin = 0.030
Mean gap = +0.008
Task-risk difference: EcoCert-V2X − A5
0.035
0.030
0.025
0.020
0.015
0.010
0.005
0.000
−0.005
S0
S1
S2
S3
S4
S5
S6
S7
Confirmatory scenario
